# Faculty Recruitment, Retention, and Representation in Leadership: An Evidence-Based Guide to Best Practices for Diversity, Equity, and Inclusion from the Council of Residency Directors in Emergency Medicine

**DOI:** 10.5811/westjem.2021.8.53754

**Published:** 2022-01-03

**Authors:** Dayle Davenport, Al’ai Alvarez, Sreeja Natesan, Martina T. Caldwell, Moises Gallegos, Adaira Landry, Melissa Parsons, Michael Gottlieb

**Affiliations:** * Rush University Medical Center, Department of Emergency Medicine, Chicago, Illinois; † Stanford University School of Medicine, Department of Emergency Medicine, Stanford, California; ‡ Duke University School of Medicine, Division of Emergency Medicine, Durham, North Carolina; § Henry Ford Health System, Department of Emergency Medicine, Detroit, Michigan; ¶ Harvard University School of Medicine, Division of Emergency Medicine, Boston, Massachusetts; || University of Florida School of Medicine, Department of Emergency Medicine, Jacksonville, Florida

## Abstract

Improving the recruitment, retention, and leadership advancement of faculty who are under-represented in medicine is a priority at many academic institutions to ensure excellence in patient care, research, and health equity. Here we provide a critical review of the literature and offer evidence-based guidelines for faculty recruitment, retention, and representation in leadership. Recommendations for recruitment include targeted recruitment to expand the candidate pool with diverse candidates, holistic review of applications, and incentivizing stakeholders for success with diversity efforts. Retention efforts should establish a culture of inclusivity, promote faculty development, and evaluate for biases in the promotion and tenure process. We believe this guide will be valuable for all leaders and faculty members seeking to advance diversity, equity, and inclusion in their institutions.

## BACKGROUND

Many academic institutions are prioritizing diversity, equity, and inclusion (DEI) in an effort to improve the recruitment, retention, and leadership advancement of faculty who are under-represented in medicine (UIM)*.^1^,^2^ Diversity in leadership provides many benefits, including the ability to reduce implicit bias in care, allow for diversity of thought and perspectives in institutional-level decisions, and improve visibility of UIM faculty who are frequently overlooked and under-represented in positions of power.^3^ Other benefits include understanding of social justice implications and improved student outcomes in the areas of professionalism, humanism, and cultural competency.^4^,^5^ In emergency medicine (EM), where diverse pathology, patient populations, and workflows are inherent, DEI efforts are a vehicle toward excellence in patient care, research, and health equity.

In medical education, lack of a diverse faculty can impede residency recruitment efforts for UIM candidates.^6^–^8^ Programs that demonstrate diversity through a higher percentage of UIM faculty had higher proportions of UIM residents.^7^,^8^ However, recent data on residents from the 20 largest specialties over 11 academic years (2007–2018) found that no specialty represented either the Black or Hispanic populations comparable to the overall United States population.^9^ In light of the critical role that faculty diversity plays in resident recruitment, optimizing patient care, and workplace culture, we sought to summarize the current literature and provide best practice recommendations for faculty recruitment, retention, and representation in leadership.

## CRITICAL APPRAISAL

This article is the seventh in a series of evidence-based best practice reviews from the Council of Residency Directors in Emergency Medicine (CORD) Best Practices Subcommittee.^10^–^15^ With the assistance of a medical librarian, we searched MEDLINE via PubMed for articles published from inception to January 21, 2021, using robust and sensitive keyword variations that relied on PubMed’s automatic term-mapping to apply the appropriate medical subheadings terms focused on diversity, equity, and inclusion (Appendix). We also reviewed the bibliographies of all relevant articles for additional studies. Articles were screened independently by two authors to evaluate for any papers addressing recruitment and retention for faculty. We included articles if either author recommended inclusion.

The search yielded 2080 unique articles, of which 70 were deemed to be directly relevant for inclusion in this review. When supporting data was not available, recommendations were made based upon the authors’ combined experience and consensus opinion. The level and grade of evidence were provided for each best practice statement according to the Oxford Centre for Evidence-Based Medicine criteria (Tables 1 and 2).^16^ Prior to submission, the manuscript was reviewed by the entire CORD Best Practices Subcommittee. It was subsequently posted to the CORD website for two weeks for review and feedback from the entire CORD community.

## RECRUITMENT STRATEGIES

### Institutional Mission Statements

Diversity and inclusion should be part of every institution’s mission statement to provide evidence of the explicit commitment to these principles as well as its importance to the advancement of health equity for the community.^1^,^17^ Institutions need to be authentic to a mission of diversity and inclusion with action, not just rhetoric.^18^ One radiology department created a departmental diversity web page as part of their recruitment efforts, which included an explicit statement of their diversity mission and videos from program leadership.^19^ The University of Michigan tied diversity to its mission of academic excellence (referred to as the “Michigan Mandate”) and allocated 1% of the university’s budget annually for diversity initiatives. As a result of this, UIM matriculation doubled, UIM faculty markedly increased, and more UIM faculty were promoted to leadership positions and received tenure.^20^

### Expand the Candidate Pool

Networking and peer support from other UIM faculty are essential to decreasing the sense of isolation and increasing satisfaction among UIM faculty.^1^,^21^,^22^ A scoping review noted that the lack of a “critical mass” of UIM faculty was a deterrent to new UIM faculty applicants, further perpetuating the imbalance.^18^ Modeled after the Rooney Rule in National Football League policy, one program required the inclusion of at least two qualified UIM candidates representing diversity in the applicant pool for each position and invited at least one of these candidates to participate in an on-campus interview.^23^ In cases where there is little faculty diversity, the 2008 CORD Academic Assembly Diversity Workgroup recommended expressing that you are actively recruiting for diverse racial and ethnic backgrounds as well as using the institution’s local and community demographics to highlight the diversity of the patient population.^24^ A 2011 survey found that medical student diversity was the strongest predictor of faculty diversity, highlighting the need to establish early pipelines and pathways.^25^ The Center for Multicultural and Community Affairs (CMCA) at the Mount Sinai School of Medicine created a dedicated council in 2008 to improve coordination of outreach, recruitment, and retention activities of UIM physician and non-physician scientists by including representatives institution-wide to support efforts from pre-matriculation through postgraduate training.^26^

### Create Diverse Recruitment Committees

Recruitment committees should be composed of a diverse group of members and/or institutional diversity leaders (eg, Chief Diversity Officer, Assistant Dean of Diversity).^1^ Diversity recruitment should be a joint effort between UIM and non-UIM faculty so that it does not unduly burden UIM faculty.^18^,^22^ Responsibilities of a diversity-oriented recruitment committee are outlined in Table 3.^1^ As part of a successful, multifaceted strategic plan to promote diversity at the University of Michigan Department of Surgery, a standing departmental recruitment committee was selected via nomination. Members were intentionally selected to ensure diversity with respect to gender, race, academic rank, and subspecialty. The committee identified a diverse pool of applicants that had been previously overlooked while maintaining faculty excellence.^23^

Similarly, the Mount Sinai Diversity Leadership Council was established to promote diversity in faculty recruitment, retention, and development. Senior-level faculty representatives (Diversity Liaisons) from all departments were chosen to enhance faculty diversity and report diversity metrics (eg, trends, climate, faculty mentoring, advancement) to the Dean. They also developed specific departmental action plans under the guidance of department chairs and shared best practices for improving faculty diversity, retention, development, and advancement.^26^

### Incentivize Stakeholders and Create Accountability

Tracking institutional and departmental diversity metrics is necessary to set goals, identify effective strategies and opportunities for improvement, and incentivize success.^26^,^27^ This process could include tracking promotion, retention, and leadership positions among UIMs vs non-UIMs.^27^,^28^ This could also include assessing the climate of inclusion with surveys, interviews, and focus groups to measure the prevalence of bias and discrimination and to document continued challenges, microaggressions, and other barriers to an inclusive workplace culture.^27^–^29^ Institutional and departmental dashboards should include diversity and equity goals to monitor performance.^1^,^30^,^31^

Incentive bonuses, academic promotion, and eligibility to leadership positions for all faculty could be tied to participation in diversity and inclusion activities and performance metrics on diversity outcomes.^32^ The Medical University of South Carolina developed an assessment tool for each department that included quantitative and qualitative variables (eg, UIM individuals recruited, grand rounds/seminars on diversity, UIM speakers, activities related to healthcare disparities and social determinants of health, and implementation of cultural competency training). Department chairs were required to complete the assessment annually, develop annual diversity goals, and report the results institution-wide, with end-of-year incentives tied to their results.^33^

External reporting of departmental and residency diversity compared with national data can be useful to rally support for recruitment resources.^17^,^34^ Funding agencies (eg, the National Institutes of Health [NIH]) could consider an institution’s demonstrated commitment to diversifying faculty when making funding decisions, particularly for diversity fellowships and grants.^35^

In the United Kingdom, the National Health Service (NHS) adopted a Workforce Race Equality Standard for all NHS organizations, requiring that they meet measurable improvement on nine diversity metrics, including adequate representation of UIM staff and senior leadership, UIM representation on organizational boards that reflect the demographics of the community, reductions in reports of discrimination, and annual open publication of progress.^36^ This led to reduced discrimination reports and improvements in UIM promotion.^37^ The Athena Scientific Women’s Academic Network was created to increase representation and equity for women in science, technology, engineering, and medicine.^38^ Institutions that improved gender parity were given awards, and in 2011 government funding from the National Institute for Health Research was restricted to those institutions.^38^ This restriction of government funding led to improvements in career satisfaction, job opportunities, and professional development.^38^ A similar model could be used for UIM faculty.

### Inclusive Marketing and Targeted Recruitment

Language, images used for marketing, and the process of disseminating promotional materials should be assessed for bias and barriers to UIM recruitment and include clear non-discrimination policies.^27^ Links to diversity and inclusion web pages at the program, departmental, and institutional levels can be used to highlight current successes and future goals.^24^,^34^ Openings should be posted on the job sites of societies representing under-represented groups (e.g., National Hispanic Medical Association).^23^ Social media can also be used to emphasize the institution’s commitment and progress in DEI efforts and engage potential candidates.^23^,^39^ Aggressive recruitment and hiring of competitive UIM candidates, even when the department is not engaged in an official search, can establish a culture that prioritizes resource allocation to faculty diversity.^31^,^40^ Department chairs should use the network of diverse faculty, diversity committee members, and national conferences to identify potential candidates.^40^

### Recruitment Packages

Existing debt, compounded by salary inequities and lower rates of generational wealth, can impact career choices by UIM physicians.^40^–^42^ One study found that UIM faculty were more likely to report needing to supplement their income vs non-UIM faculty.^41^ This has led some UIM faculty to pursue non-academic positions and UIM physicians with significant debt to have greater attrition rates.^21^,^41^,^43^ This suggests that debt reduction programs, which benefit all faculty, may result in reduced attrition rates for UIM faculty.^41^ In fact, institutions should consider targeted funding initiatives and recruitment packages specifically for UIM faculty,^17^,^44^ as medical school recruitment packages (eg, salaries, research and development resources, flexible work hours, and environment that promote growth and success) were found to be the primary factor in the recruitment of UIM faculty.^31^ In 2004, one otolaryngology department created a multifaceted effort to actively recruit and retain diverse faculty, which included an evaluation of salary.^45^ Over a 10-year period, they saw a significant increase in the percentage of women and UIM faculty, as well as the resolution of salary differences for women.^45^

### Holistic Review, Standardization, and Faculty Ambassadors

Faculty selection should employ a holistic review of candidate applications.^19^,^30^ Holistic review emphasizes the need to assess characteristics that the institution values. For example, one group asked questions that were behavior-based on topics related to clinical practice, education, leadership, and diversity and inclusion (eg, “What do you see as the fundamental characteristics of an inclusive environment?”).^23^ They also sought to standardize the interview process by conducting group interviews for each candidate, having the same committee member ask the same question of all candidates, and using a standardized evaluation tool and scoring system.^23^ Another group used faculty ambassadors, which connected a current UIM faculty member with faculty recruits from various departments during the interview process. The ambassador shared their experiences, discussed the work environment, the community, and social opportunities.^33^,^46^ Following the interview, all candidates, hires, and committee participants should be asked to assess the overall strategy and provide feedback of the program.^23^,^47^

### Implicit Bias Training

Interviewees are subject to biases of the interviewer, particularly when assessing the “fit” of a candidate.^48^ This can be particularly problematic for women and UIM candidates, with one study finding that fictitious resumes of Black candidates were rated more negatively than those of White candidates.^49^ In surgical and procedure-based disciplines, even the evaluation of technical skills is subject to bias, impacting recruitment and advancement.^23^ Therefore, it is important to engage in anti-bias training for interviewers,^27^,^50^ with one program requiring its recruitment committee to complete the Implicit Association Test and an Association of American Medical Colleges (AAMC) online unconscious bias seminar.^23^

**Best Practice Recommendations**

1. The institutional and departmental mission statements should include an explicit commitment to diversity, equity, and inclusion. (Level 5, Grade D)
2. Institutions and departments should make focused efforts to expand the candidate pool with diverse candidates. (Level 4, Grade C)
3. Departmental and institutional recruitment committees should include diverse membership. (Level 4, Grade C)
4. Institutions should incentivize all stakeholders and increase accountability for diversity efforts. (Level 3b, Grade C)
5. Departments and institutions should engage in inclusive marketing and targeted recruitment of UIM candidates. (Level 3b, Grade C)
6. Institutions should consider recruitment packages and debt reduction programs for UIMs and ensure equitable salaries (Level 3b, Grade C)
7. Interview committees should use a holistic review of applications and consider faculty ambassadors. (Level 3b, Grade C)
8. Interviewers should undergo implicit bias training. (Level 4, Grade C)

## RETENTION STRATEGIES

### Establish a Culture of Inclusivity

Improving diversity cannot occur without creating a climate of inclusion that promotes cultural understanding and cultural competency.^1^,^33^,^51^ The AAMC outlines a four-step process for assessing an institution’s culture with reflective questioning, data collection, synthesis and analysis to identify barriers, and the creation and assessment of outcomes (Table 4).^52^ Facilitated discussions on race and racism can create constructive dialogues to reduce prejudice and misinformation.^1^,^22^ Faculty should undergo organization-wide training to identify and respond to structural racism, address personal biases (via implicit association testing and bias training), and have pathways of accountability for intolerance and discriminatory behaviors through effective formal channels (eg, human resources, supervisors, ombudsman).^18^,^29^,^30^,^53^ The system must support individuals subjected to discrimination and reporters of discrimination who fear retaliation. 29 Institutional policies must also combat structural racism in evaluations, compensation, promotions, and leadership opportunities including annual reviews to assess for bias.^22^,^29^,^54^

### Address Unique Burdens of UIM Faculty

Faculty who are UIM experience differential treatment secondary to their race and ethnicity, impacting wellness, mental health, academic productivity, and increasing turnover.^1^,^42^ They also describe feeling increased scrutiny and the need to represent the entirety of their race/culture with a pressure to be near-perfect in both clinical and non-clinical environments.^22^ The lack of inclusion and recurrent microaggressions they experience or witness causes feelings of stress, anxiety, hopelessness, social isolation, and expendability.^1^,^29^,^55^ To combat these, some experts have recommended wellness initiatives that specifically address the unique experiences and challenges of UIM faculty.^22^,^42^

These UIM faculty, especially junior UIM faculty, are often disproportionately asked to participate in administrative/committee responsibilities, volunteer in community settings, and mentor UIM students or residents relative to non-UIM faculty.^18^,^43^,^56^ Although it is helpful for the institution to have UIM role models for trainees, this can undermine UIM faculty success and career development by decreasing the time available to participate in scholarly work that is more valued (eg, grants, publications) while balancing clinical work.^1^,^40^,^57^,^58^ Diversity initiatives should not impose a “tax” on UIM faculty, but should be an institutional goal where all administrators and leaders are trained to recognize and address biases and are responsible for implementing diversity and inclusion initiatives. Educational value units or equivalent credit should also be created to recognize and reward diversity work via non-clinical time, career advancement, and financial compensation.^31^

While diversity has been lauded as a means to decrease healthcare disparities and provide a pool of physicians to care for underserved patients, UIMs should not be selectively steered or expected to care for underserved populations. The medical workforce as a whole should share responsibility for meeting the healthcare needs of the underserved. The repetitive mention in the literature of UIM physician service commitment to vulnerable populations reinforces a narrative that may limit UIM practice, research, and leadership opportunities.^4^ Similarly, the argument for physician-patient concordance creating better healthcare outcomes may limit UIM physicians’ ability to practice in all regions and may lead to the perception that similar benefits would be seen with non-UIM physicians (eg, White patients would receive better care from White physicians).^4^ Therefore, it is important to support UIM faculty interests and avoid making assumptions about their preferred patient populations or fields of research.

### Institutional Diversity Leaders

Many institutions have introduced a Chief Diversity Officer (CDO), whose responsibilities include initiating, developing, and ensuring compliance with institutional and federal diversity strategies. The CDO may also promote health equity research, ensure equitable sourcing of vendors, support affinity marginalized groups, and address disparities in the patient experience.^31^ To be effective this individual must have the power and influence to enact change.^59^ Creating a CDO position without institutional diversity efforts has not been shown to significantly impact faculty diversity.^60^ In addition to the CDO position, institutions should consider assistant/associate deans of diversity. The Medical University of South Carolina created a diversity office staffed by a senior associate dean for diversity, an associate dean for resident inclusion and diversity education, a manager for recruitment, and a manager for diversity initiatives.^33^ A radiology department created a Vice Chair of Diversity as part of its efforts to increase diversity. They also collaborated frequently and directly with the institution’s Office of Diversity and Associate Vice Dean of Diversity.^19^

### Leadership and Academic Advancement

While racial/ethnic minorities consist of 40% of the US population, UIM physicians comprise only 9% of medical school faculty and 18% of medical students.^3^,^61^ Although data from the AAMC shows that UIM representation has increased over time, UIMs are less likely than their non-UIM colleagues to be promoted from assistant to associate professor and from associate to full professor.^42^,^62^,^63^ Over a 10-year period, the probabilities of promotion were lower and probabilities of attrition were higher for UIM faculty and women.^41^ UIM physicians are less likely to hold administrative leadership positions in various departments,^3^,^64^–^66^ serve as program directors,^67^,^68^ receive NIH research awards,^3^ grants,^26^ and receive tenure^69^ than their non-UIM peers. Even after adjusting for tenure status, degree, gender, and NIH award status, UIM faculty have significantly longer time to promotion when compared to their White counterparts.^1^ Therefore, it is critical to ensure UIMs are advanced equitably and to assess for bias in the promotion and tenure process. Transparency regarding the criteria for promotion, a systematic plan to address disparities in promotion, and consistent mentoring of UIM faculty to meet these criteria is also necessary.^1^ Additionally, UIM faculty should be sought out for new leadership positions and all institutions should prioritize a diverse leadership team.^69^,^70^

### Faculty Development Programs

A 2012 study found that only 29% of medical schools had faculty development programs specifically targeted to UIM faculty.^62^ Effective faculty development programs should be institution-wide, rather than just select departments and divisions.^17^ One institution created an institution-wide diversity program with structured individual mentoring to UIM faculty, specific professional development opportunities, social events, and salary support for scholarly endeavors, which led to an increase in the percentage of UIM faculty from 4% to 7%.^62^ Another institution initiated a strategic plan to increase diversity among its students, resident physicians, and faculty, which involved an expansion of pipeline and mentoring programs (ranging from high school to faculty), and nearly doubled the number of UIM faculty.^33^ One medical school sponsored educational programs for faculty leadership development, including programs specific to UIM faculty resulting in a doubling of the number of UIM faculty.^71^

The Harold Amos Medical Faculty Development Program (AMFDP), a national program of the Robert Wood Johnson Foundation, was instituted to support academic physicians from historically disadvantaged backgrounds to promote faculty diversity and address health inequity. In a study using the 2003–2008 application period, scholars (individuals who were funded by AMFDP) and non-scholars (individuals who completed final-round interviews but were not funded) were compared. Scholars and non-scholars had similar levels of academic productivity with no differences in publications, federal grant awards, or federal grant dollars. However, scholars were more likely to report attaining a leadership position, earning a promotion to associate professor or higher, and remaining in academic medicine.^3^

Institutional support to help UIM junior faculty, particularly clinician-investigators, can be accomplished by creating internal faculty development programs, institutional minority faculty development awards, and salary support/protected time as the faculty member awaits independent funding.^40^ Programs should have formal didactics on teaching, manuscript writing, preparation of grant applications, leadership, and training in additional areas critical for research (eg, biostatistics).^31^,^35^ Guidance on negotiation, grants management, mentoring, and work-life balance is also beneficial.^3^ The Mount Sinai CMCA established a Faculty Scholars Program that engaged over 60 junior faculty in formal research training and academic development programs. Fourteen of the scholars ultimately participated in a sponsored Master of Science in Clinical Research or Master of Public Health program.^26^

### Local Mentorship and Sponsorship

A qualitative study of UIM pediatric emergency physicians underscored the need for early mentorship and opportunities to enter a leadership pathway.^22^ However, there has been a dearth of UIMs in academic medicine to serve as mentors and role models.^21^ Both UIM and non-UIM faculty should receive mentor training and serve as mentors for UIMs.^32^ Another qualitative study of Black students emphasized the importance of having both UIM and non-UIM mentors.^55^ In the strategic plan for diversity by the Medical University of South Carolina, each department developed a mentoring plan, identified a mentoring champion as a liaison to the Dean’s Office, and paired junior faculty members with senior faculty for academic and professional development.^33^ Senior faculty are instrumental for mentorship, and inclusion on grants can help advance a UIM junior faculty’s career and potential for future grants.^43^ Jeffe et al found that mentored K awardees had higher rates of retention and promotion. There was also a greater likelihood of promotion among assistant professors who received NIH awards.^41^ Therefore, it is important to support and mentor UIM clinician-researchers.

Non-UIM faculty more commonly receive sponsorship (senior members who amplify and promote junior members) than UIM faculty.^18^ In a study of women faculty in medicine, women were less likely to be nominated for awards or new positions with UIM women most negatively impacted. The authors posited that social circles and familiarity created a perpetual culture of nominating the same White males for opportunities.^72^ Thus, diversity councils and diversity champions who have both resources and influence are necessary to support UIM faculty retention and career advancement.^18^

### Promote National Organization Membership

In semi-structured interviews of women in academic emergency medicine at various stages of their careers, active participation in a national, woman-focused organization was found to engender opportunities and relationships that facilitated leadership. Membership increased access to mentors and sponsors, enabled scholarly work via peer mentorship and collaboration, assisted with navigating through barriers and bias, presented opportunities for awards, recognition, and speaking engagements, and cultivated a sense of belonging.^73^ Similarly, a survey of members in the Academy for Diversity and Inclusion in Emergency Medicine within the Society for Academic Emergency Medicine found that participation led to more publications, didactic presentations, grand round presentations, and mentor/mentee relationships.^74^ The Association of Black Cardiologists aims to promote diversity, boost collegiality in the field, and promote health disparities research and interventions. Through its scholarships, this group was able to partially fund cardiology subspecialty training for 44 Black cardiologists.^75^

The Academic Pediatric Association (APA) Research in Academic Pediatrics Initiative on Diversity (RAPID) is sponsored by the NIH National Institute of Diabetes and Digestive and Kidney Disorders. It is the first research-education program aimed at the successful recruitment, retention, and professional advancement of diverse early-career faculty in general academic pediatrics who are pursuing research careers. The RAPID key components include small research grants for young investigators, mentoring with faculty from the National Advisory Committee, networking at the annual Pediatric Academic Societies meeting, and career development at the annual two-day RAPID conference. The conference discusses research skills such as grant writing, publishing, presenting at national meetings, recruiting minority patients, and implicit bias, and addresses the unique challenges of minority faculty. There are also monthly conference calls to discuss research progress as part of a peer network. These RAPID scholars produced 56 publications and presented nationally. Participants felt the program helped them attain additional funding, NIH awards, and grants, and amplified their career trajectory. As participants were required to join the APA, it also increased the diversity of the national organization.^76^

**Best Practice Recommendations**

1. Establish a culture of inclusivity. This should include cultural competency and bias training, as well as initiatives to identify and address discrimination. (Level 3b, Grade B)
2. Avoid overusing UIM faculty for administrative and mentoring positions and ensure that UIM faculty are properly supported and recognized for their contributions. (Level 3b, Grade C)
3. Create institutional diversity leadership positions, such as a Chief Diversity Officer or Assistant/Associate Dean of Diversity, that are backed by institutional support. (Level 3b, Grade C)
4. Ensure UIM faculty are promoted appropriately and evaluate for biases in the promotion and tenure process. (Level 3b, Grade C)
5. Create faculty development programs specifically focused on UIM faculty. (Level 3b, Grade B)
6. Pair UIM faculty with both UIM and non-UIM mentors. Leaders should sponsor qualified UIM faculty for opportunities. (Level 3b, Grade C)
7. Promote and support engagement with national organizations. (Level 3b, Grade B)

## LIMITATIONS

While we used a comprehensive search methodology, it is possible some pertinent articles may have been missed in the current review. However, we sought to minimize the risk by reviewing all related studies in the bibliographies of included articles, using content and topic experts and undergoing pre-submission review and approval by the CORD community. Another limitation is the paucity of interventional studies and those focused on EM specifically. When robust, EM-specific data was not available, we used studies from other medical specialties, health-related professions, and expert opinions. Thus, some proposed recommendations may not be as effective for EM, and further studies are needed to ensure pertinence to EM.

## CONCLUSION

Recruitment, retention, and advancement of UIM faculty are critical to increasing diversity, equity, and high-quality clinical care and trainee education within emergency medicine. This paper summarizes key strategies and provides best practice recommendations. We hope this manuscript will inform readers on how best to promote each of these components.

## Supplementary Information



## Figures and Tables

**Table 1:** Oxford Centre for Evidence-Based Medicine Levels of Evidence.^16^

Level of evidence	Definition
1a	Systematic review of homogenous RCTs
1b	Individual RCT
2a	Systematic review of homogenous cohort studies
2b	Individual cohort study or a low-quality RCT*
3a	Systematic review of homogenous case-control studies
3b	Individual case-control study**
4	Case series or low-quality cohort or case-control study***
5	Expert/consensus opinion

* defined as <80% follow up;

** includes survey studies and cross-sectional studies;

*** defined as studies without clearly defined study groups.

RCT, randomized controlled trial.

**Table 2:** Oxford Centre for Evidence-Based Medicine grades of recommendation.^16^

Grade of evidence	Definition
A	Consistent level 1 studies
B	Consistent level 2 or 3 studies or extrapolations* from level 1 studies
C	Level 4 studies or extrapolations* from level 2 or 3 studies
D	Level 5 evidence or troublingly inconsistent or inconclusive studies of any level

* ”Extrapolations” are where data is used in a situation that has potentially clinically important differences from the original study situation.

**Table 3:** Responsibilities of recruitment committees.^1^

Define diversity criteria for potential candidates. Create a diversity statement. Implement a strategic process for recruitment of diverse candidates. Monitor the success of the recruitment initiatives. Advocate for change throughout the institution.

**Table 4:** American Association of Medical Colleges four-step process* for assessing institutional culture.

Step 1: Reflective Questions	Begin the process of understanding diversity and inclusion in your institution by personal reflection on relevant criteria.
Step 2: Data Collection	Gather qualitative and quantitative indicators of diversity and inclusion at your institution.
Step 3: Synthesis and Analysis	Carefully identify the areas of strength and opportunities for development at your institution.
Step 4: Leverage Findings	Translate the products of your assessment into institutional outcomes through communication with stakeholders and institutional change agents.

* Adapted from AAMC.^52^

## Footnotes

* Under-represented minority (URM) was a term defined by the Association of American Medical Colleges (AAMC) consisting of Black, Mexican-American, mainland Puerto Rican, and Native American (referring to American Indian and natives of Alaska and Hawaii) racial groups. In 2004 the AAMC expanded this definition to “under-represented in medicine” (URiM or UIM); UIM references those ethnic and racial populations in the medical profession who are under-represented relative to their respective numbers in the greater population. For consistency, we will use the term UIM, although we acknowledge the variable usage both in literature and in practice.

## Appendix Search strategy

(((medical education OR meded[tiab]) AND (recruitment OR recruit* OR retention[tiab] OR retain* OR pipeline)) AND (diversity OR diverse OR inclusive OR underrepresented OR minority OR minorities OR ethnic OR ethnicity OR ethnicities OR racial OR race OR tokenism OR token[tiab] OR Black OR Asian OR Blacks OR Asians OR Puerto Rican OR Mexican American OR Native American OR American Indian OR Alaskan Native OR Hawaiian OR African American OR Hispanic OR Latino OR Latinx OR Latina)) AND (physician OR doctor OR trainee OR residency OR trainees OR residency OR interns OR intern OR faculty)
